# A Surgical Case of Bow Hunter's Syndrome Diagnosed by Cervical Rotational MRA

**DOI:** 10.1155/2022/6091597

**Published:** 2022-08-04

**Authors:** Hidenori Matsuoka, So Ohashi, Michihisa Narikiyo, Ryo Nogami, Keita Hashimoto, Hirokazu Nagasaki, Yoshifumi Tsuboi

**Affiliations:** Department of Neurosurgery, Kawasaki Saiwai Hospital, 31-27 Omiyacho Saiwai-Ku Kawasaki Kanagawa 212-0014, Japan

## Abstract

Bow hunter's syndrome is an ischemic manifestation of vertebrobasilar artery (VA) insufficiency due to stenosis or occlusion of the contralateral VA at the bony elements of the atlas and axis during neck rotation. In early reports, VA stenosis at the craniovertebral junction was the main cause, but later, symptoms due to VA occlusion at the middle and lower cervical vertebrae were also included in this pathology. Although the confirmed diagnosis is usually determined by dynamic digital subtraction angiography (DSA), we have experienced a method of minimally invasive MR angiogram (MRA) that provides the same diagnostic value as DSA and would like to present it here. The patient was a 61-year-old man who had been visiting the outpatient clinic for cervical spondylosis due to neck pain for 9 months. When he rotated his neck to the left side, dizziness and syncope appeared. Initial MRA in the neutral position did not show any steno-occlusive changes in the vertebrobasilar artery. In our hospital, repeated MRA with the neck rotated 45 degrees to the left demonstrated ipsilateral left VA severe stenosis. Subsequent DSA showed the same findings, with occlusion of the left VA. CT of the cervical spine revealed a ventral C3/4 osteophyte within the foramen. Based on these findings, instability at the C3-4 during head rotation was considered the cause of the vertebrobasilar insufficiency. The patient underwent anterior discectomy and fusion (ACDF) at the C3/4 level using a cylindrical titanium cage. Immediately after the surgery, the patient's symptoms improved dramatically and did not appear even when the neck were fully rotated to the left. More than 5 years have passed since the surgery, and the patient is still in good health.

## 1. Introduction

Bow hunter's syndrome is a condition in which the vertebral artery (VA) is stenosed or occluded at the craniovertebral junction during neck rotation, causing ischemic symptoms in the vertebrobasilar artery circulation [1, 2]. In early reports, VA stenosis at the craniovertebral junction was the main cause, but in recent years, symptoms due to VA occlusion at the middle and lower cervical portion have been included in this pathological condition, and it is now called rotational vertebral artery occlusion (RVAO) [3, 4]. Physiological compression of bones, osteophytes, and ligaments during rotation of the neck can induce occlusion of the VA [3]. Although blood flow in the VA in the neutral position is normal, it is mostly hemodynamic mechanisms that induce occlusion and dissection of vessels in the VA when the neck is rotated. For the diagnosis of RVAO, digital subtraction angiography (DSA) is usually used to obtain a definitive diagnosis. There have been reports of less invasive diagnostic methods such as computed tomographic angiography (CTA), ultrasound, and ultrasound-guided CTA [5] that can evaluate dynamic blood flow in real time. The diagnostic method we performed this time was confirmed by MRA with the neck rotated moderately until just before the onset of symptoms. There have been various reports on surgical treatment options for this pathological condition, such as posterior fixation, direct anterior decompression surgery, and endovascular treatment, but so far no consensus has been reached on which treatment is best [6].

## 2. Case Presentation

**Figure 1:**
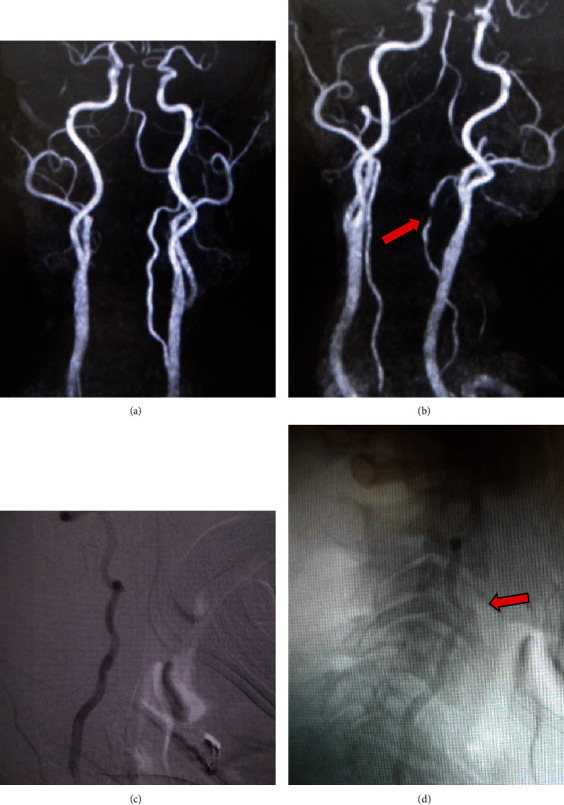
Preoperative MRA shows normal flow and left VA dominant in the neutral position (a). MRA with neck rotation of 45 degrees revealed severe stenosis (arrow) of left VA (b). Preoperative cerebral angiograms of left VA show (c) normal flow in the neutral position and (d) severe stenosis at C3-4 level (arrow) and decreased flow on leftward neck rotation.

**Figure 2:**
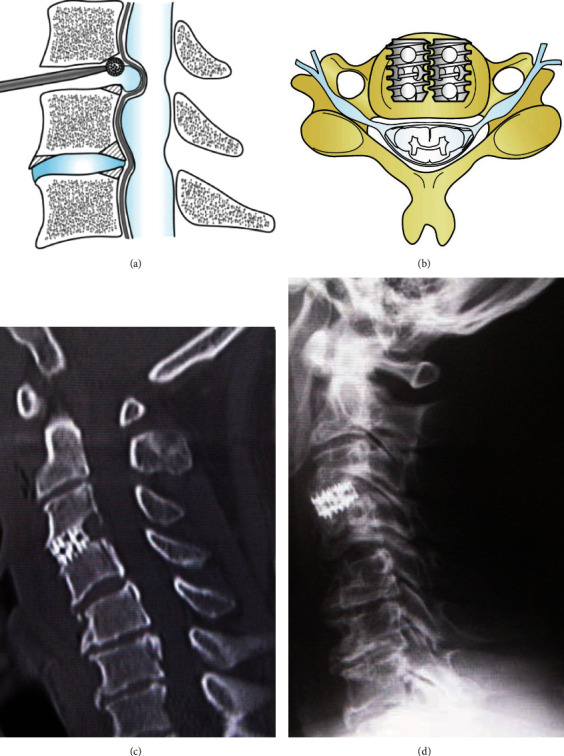
Schematic drawings in the sagittal view of anterior cervical discectomy and fusion (ACDF) and postoperative CT. Osteophytes after discectomy are drilled out by a diamond burr (a). Cages are inserted into the disc spaces in the twin-cage and locking fashion after decompression. Postoperative (c) CT and (d) X-ray show sufficient decompression, and the cage was inserted in the optimal position.

A 61-year-old man had been suffering from neck pain for about 9 months and was diagnosed with cervical spondylosis at the previous outpatient clinic. After that, he noticed dizziness during neck rotation to the left, and maximal rotation of the neck led to unconsciousness. MRI demonstrated no intracranial ischemia, and MRA in the neutral position showed no stenotic changes of the VA, and the left VA was dominant compared with contralateral VA. MRA with neck rotation of 45 degrees clearly illustrated severe stenosis of the ipsilateral VA at the midline cervical portion. At this time, the left-sided VA was generally poorly defined, and the contralateral right-sided VA was better visualized. The DSA was performed later for a definitive diagnosis, and the results were the same as the MRA results (Figure 1). A subsequent CT of the cervical spine revealed a ventral C3/4 osteophyte within the foramen. In response to the above findings, we diagnosed ischemic symptoms due to obstruction of the left VA during neck rotation due to C3-4 osteophytes. We concluded that the cause was that the osteophytes physically pressed the left VA due to instability of C3-4 during neck rotation and selected the surgery of anterior cervical discectomy and fusion (ACDF). After removing the intervertebral disc at C3/4, the osteophytes were drilled with a diamond burr and fixed with titanium cylindrical cages (Figure 2). The patient's symptoms improved dramatically after the surgery, and no symptoms appeared even when the neck was rotated to the left as much as possible. Even now, more than 5 years after the surgery, no recurrence of symptoms has been observed.

## 3. Discussion

RVAO is a relatively rare disease that often develops with symptoms such as dizziness, vertigo, and syncope due to vertebrobasilar artery insufficiency [1–3]. Previous report indicates that 75% of patients with RVAO had embolic stroke [7]. RVAO in the middle and lower cervical vertebrae is ineffective for conservative treatment due to physiological compression of bones, osteophytes, and ligaments, and surgery tends to be the best treatment option in most cases. This condition can be clearly diagnosed by DSA using contrast media, but it also has the drawback of being invasive and not applicable to all patients. In addition, some complications have been reported in DSA, including serious ones such as acute renal failure, contrast medium allergy, and cerebral infarction. The greatest advantage of dynamic DSA is that it can detect the exact moment of VA occlusion by simultaneously performing angiography and neck rotation. Therefore, obtaining a definitive diagnosis is assured. Although CTA can clarify the relationship between the artery and the surrounding bone tissue, the volume of contrast medium is too large and multiple imaging is unfeasible. Both DSA and CTA are disadvantageous in terms of radiation dose, contrast media, and invasiveness. Ultrasonography is representative of noninvasive and has been reported to be useful in the diagnosis of RVAO, but it has disadvantages such as the possibility of differences in diagnosis depending on the skill of the technologist and sufficient inspection time is required. On the other hand, we would like to propose MRA, which is the simplest and most noninvasive examination and can be performed by anyone, because it can be diagnosed by simply rotating the neck. The most important factor is that the patient should not rotate the neck until symptoms appear, and MRA should be performed with moderate rotation. If the neck had been rotated to the maximum, it would not have been possible to maintain a rest and a clear image could not be obtained. In the present case, a comprehensible image was obtained by rotating the neck 45 degrees to the left. Rotational movement of the neck is the key to this condition, but cases of VA obstruction in which symptoms appear in the extended position of the neck have also been reported [8]. In this case, an MRA with the neck extended may be useful for early diagnosis. In the future, MRA with moderate cervical rotation will be useful for diagnosing patients with contrast medium allergies and severe renal dysfunction, which are contraindicated for DSA. We believe that this case report proved that moderate rotational MRA could be a reliable and less invasive diagnostic tool.

The patient underwent C3-C4 anterior cervical discectomy and fusion without vertebral artery decompression. This case report shows that the obstruction of the vertebral artery due to cervical rotation can be successfully managed using the anterior approach without direct decompression. Currently, there is no consensus on the treatment of RVAO [6], and anterior decompression and endovascular treatment have been reported [9]. In the case of direct anterior decompression, surgery may be difficult due to bleeding from the veins around the VA. In addition, evaluation methods such as intraoperative DSA and indocyanine green have been reported to confirm the blood flow of VA after direct decompression [10, 11]. The anterior approach without decompression is slightly safer than the direct anterior approach, suggesting a lower risk of vertebral artery injury. ACDF is a standard treatment with established long-term results used for degenerative diseases [12], and we are confident that it will provide a safe and viable alternative for the treatment of symptomatic RVAO.

## 4. Conclusion

MRA in the cervical rotation, which is a relatively simple test for symptomatic RVAO, helped in the early diagnosis. Treatment was remission of symptoms by selecting ACDF, which has been established as the standard treatment for degenerative diseases.
